# Understanding financial hardship and financial recovery among clients in supported accommodation services

**DOI:** 10.1371/journal.pone.0334211

**Published:** 2025-10-09

**Authors:** Wendy M. M. Albers, Jaap van Weeghel, Diana P.K. Roeg

**Affiliations:** 1 Research and innovation, Kwintes Supported Housing, Zeist, Netherlands; 2 Tranzo Scientific Center for Care and Wellbeing, School of Social and Behavioural Sciences, Tilburg University, Tilburg, Netherlands; Otago Polytechnic, NEW ZEALAND

## Abstract

**Background:**

Financial stability is essential for well-being, influencing health, housing, education, and social participation. Individuals with mental illness often face financial hardship, including debt, financial administration problems, and limited access to basic needs. Despite its importance, little research exists on factors supporting financial self-management for this group. This study explores financial hardship experiences, causes, and support mechanisms among clients in supported accommodation in the Netherlands.

**Methods:**

Twenty-seven clients were recruited via staff of a large supported accommodation setting in the Netherlands, of which 22% received residential support and 48% received floating outreach. Semi-structured interviews were conducted, and thematic analysis was used to interpret participants’ lived experiences with financial problems.

**Results:**

Participants identified several causes for their financial struggles, including psychological issues, impulsive spending, and a lack of financial literacy. Relationship problems (e.g., divorce) and limited social support also were mentioned to contribute to ongoing financial hardship. Budget coaches, professionals with expertise in debt management, offer personalized guidance and practical assistance with financial management, including creating budgets, managing expenses, and addressing debts. This support was highly valued by clients, helping them to organize their finances, manage expenses, reduce stress, and develop their financial literacy.

**Conclusions:**

This study highlights the complex and multifaceted nature of financial difficulties experienced by clients in supported accommodation. Budget coaches, with their specialized knowledge of the target population and financial expertise, can play a crucial role in improving the financial situation of individuals with mental illness. The findings underscore the need for integrated support addressing both financial and mental health challenges to promote long-term independence and well-being for this vulnerable population in the Netherlands.

## Introduction

Financial stability is a fundamental human need that intersects with numerous aspects of well-being, including physical health, housing, education, and social connection [1–9]. Financial hardship, characterized by difficulties meeting financial obligations, inadequate housing, healthcare access, debt burdens, or financial skill deficits, is a complex issue with far-reaching consequences [10–12]. Individuals experiencing poverty or financial stress are at heightened risk of adverse health outcomes, such as cancer, diabetes, and obesity, and have reduced life expectancy compared to their more affluent peers [13]. Beyond physical health, financial hardship is associated with psychological distress, including shame, anxiety, and depression [14,15]. This financial precarity often results in a short-term mindset [16,17], limiting individuals’ ability to participate fully in society [18].

Many people with mental illness experience financial problems [1,6,10,19]. They are disproportionately reliant on government benefits, with low employment rates (15–20%) and elevated rates of debt compared to the general population (23% vs. 7.4%) [20–24]. One study indicated that even 59% of the individuals with mental illness in high-income countries do not have enough money to access to food, shelter, or medication [10]. The financial strain is exacerbated by difficulties with money management often associated with mental health conditions and poor executive functioning, including impaired planning, memory, and problem-solving abilities [25]. The relationship is reciprocal; having financial stress also places people at higher risk of developing a mental illness [5,16]. Furthermore, dealing with financial problems and the accompanying stress hampers both clinical and personal recovery [8,26].

Supported accommodation, encompassing both community-based and residential supported housing settings, focuses on recovery by integrating clients into the community and supporting them by creating daily structure, fostering independent living, and enhancing their financial stability [27–29]. Clients rely on both rehabilitation and recovery-oriented, as well as practical support from trained social workers. This practical support includes help with personal care, managing bills, cooking, cleaning, and other daily living skills [30,31]. Despite financial difficulties being a major concern for clients [32,33], with better financial management ranking as their most important personal and support goal [2–4], and unmet support needs in this area [8,34], research on the complexities of financial hardship among this population remains limited.

While research has explored the impact of financial difficulties on the identity and well-being of individuals with mental illness, and identified coping strategies employed by these individuals, there remains a lack of knowledge regarding the factors that contribute to financial self-management within this specific population. One study explored experiences of community mental health care clients in managing their finances, revealing significant challenges [19]. Participants reported struggling to meet basic needs, experiencing heightened financial anxiety, and facing the double stigma of poverty and mental illness. Despite these challenges, formal financial support mechanisms, such as debt restructuring or protected guardianship, were often perceived as insufficient, leading clients to rely on informal support networks and develop their own coping mechanisms. Other studies have highlighted the sense of ‘being stuck in life’ due to financial constraints [35]. Clients lacked the money to maintain social relationships, and employers would not hire them, leading to reliance on benefits. Research also indicates that financial hardship can negatively impact social connections and sense of self [18]. Notably, no evidence-based interventions exist specifically for clients with mental health problems in supported accommodation services. A few studies showed the potential of representative payeeship in veterans [36] or individuals with substance use disorder [37], or of money management programs, also in veterans [38,39]. A comprehensive understanding of the underlying causes and consequences of financial hardship is essential for developing effective interventions to support clients in achieving financial stability and self-management.

The present study aims to explore experiences of financial hardship of clients in supported accommodation. We focus on the factors that have contributed to financial hardship, its impact, and, importantly, what supports financial stability, financial literacy, and self-management. In line with the literature, we understand financial literacy as encompassing not only knowledge and understanding of financial concepts, but also the skills, motivation, and confidence to apply that knowledge to make well-informed financial decisions [40,41]. Financial self-management refers to budgeting, planning, managing expenses, addressing debts, and making informed financial decisions, acknowledging that for individuals with severe mental illness these abilities may fluctuate over time and require tailored support [42,43]. This qualitative study is among the few to explore this topic within this specific population and care setting, addressing a critical gap in knowledge and potentially informing the development of targeted support programs. The research question guiding this study is: How do clients in supported accommodation experience financial hardship, and what supports promote their financial stability and self-management?

## Materials and methods

### Setting

Supported accommodation services offer a range of support modalities, including housing stability, education, employment, financial management, and daily living skills, delivered by qualified professionals to individuals with severe psychosocial difficulties residing in community-based settings, either independently or in shared living arrangements [44]. In the Netherlands, these services emerged in the 1980s as an alternative to long-term psychiatric wards in mental health care institutions [45]. Initially, they offered either outpatient support or supported housing. Nowadays, around 75% of the clients receive floating outreach, while 25% lives in supported housing or shelter facilities. Since the 1980s, more intermediate forms of housing have developed, making this dichotomy somewhat artificial [28,46,47]. However, all forms of supported accommodation aim to enable independent living, with varying levels of intensity tailored to individual healthcare needs. This study was conducted within a large Dutch supported accommodation organization providing these services. This organization offers a broad spectrum of services, including accommodation-based supported housing, floating outreach, sheltered facilities for the homeless, women’s shelter facilities, and assertive outreach to over 6,500 clients across five provinces, in both rural and urban areas.

Within these services, trained social workers provide initial financial support to clients. For complex financial issues, clients are referred to so-called budget coaches. These professionals, with post-bachelor degrees in debt management, are specifically trained to address the unique challenges faced by this client group. Their role is to empower clients to achieve financial self-management through self-regulation and improved financial management skills over a two-year period, encompassing debt management and budgeting competencies. A key advantage of budget coaches embedded in supported accommodation services is their knowledge of the target population and the short lines of communication. Complementary financial support can also be provided by Dutch municipalities or private organizations [48].

### Design

Semi-structured in-depth interviews were conducted with individuals receiving financial support within the aforementioned supported accommodation organization in the Netherlands. A thematic analysis approach was employed to explore the significance of lived experiences with financial problems and their impact. This methodology allowed for a rich and in-depth understanding of participants’ perspectives and perceived needs [49]. The present study forms part of a larger research project investigating the prevalence and nature of financial difficulties among supported accommodation clients [48], which also included focus groups with staff to explore their experiences of providing financial support. We received an official exemption for the project from the rigorous review by the Medical Ethics Committee Isala Zwolle, because it was considered as not being subject to the Medical Research Involving Human Subjects Act. The study does not involve a medical-scientific research question and, although participants were asked to participate in interviews, they were not subjected to procedures nor required to follow behavioral guidelines as defined under the WMO.

### Participants and recruitment

Participants were recruited from the large Dutch supported accommodation organization providing financial support services through budget coaches or social workers. Professionals involved in the larger research project were asked to invite eligible clients to participate in the study and providing them with information letters. Additionally, we also asked to spread the information letter across their teams. Upon receiving consent from clients, their contact details were shared with the principal researcher (WA), who initiated contact via telephone or email. During these initial contacts, study participation was reconfirmed, questions were addressed, and consent forms were subsequently sent to potential participants. Scheduling of interviews followed. Non-participation had no consequences for the support they received.

Inclusion criteria encompassed individuals aged 18 years or older, willing to participate, and receiving financial support for a minimum of three months. Exclusion criteria included insufficient Dutch language proficiency and inability to complete the interview due to florid psychosis, psychiatric crisis, or prolonged psychiatric hospitalization. To ensure theoretical saturation and capture the heterogeneity of the target group, a minimum of 20 interviews was aimed for, with a focus on achieving maximum variation in terms of gender, age, and care setting.

### Data collection

All interviews were conducted by the principal researcher (WA), a senior female researcher, skilled in qualitative interviewing, between December 2020 and March 2021. She has done her PhD on victimization and social inclusion and is experienced in discussing sensitive topics with clients with psychiatric vulnerabilities. Due to the COVID-19 pandemic, interviews were conducted via telephone or Zoom, with no apparent compromise to data quality or richness [50]. All participants provided written informed consent prior to the interview. The written consent form detailed the study's objectives, procedures, potential risks and benefits, and the participants’ right to withdraw at any time. Participants signed the form to indicate their voluntary agreement to participate. Interviews lasted an average of 33 minutes (range 17–53). At the start of the interview, study objectives were again explained, participants were assured of anonymity and data confidentiality, and audio recording was obtained with participant consent, unless otherwise requested. Participants received a small token of appreciation for their time and effort following the interview. The interview guide (please see S1 Appendix) focused on the causes and impact of financial problems, as well as experiences with financial support.

### Analyses

All interviews were transcribed verbatim and pseudonymized. Data analysis was guided iteratively by the Qualitative Analysis Guide of Leuven, a ten-stage framework that prioritizes the preservation of individual narratives [51]. Initial familiarization with the data was achieved through multiple readings of each transcript. Subsequently, narrative interview reports and conceptual interview schemes were developed for each participant, closely adhering to their original language to maintain authenticity, and ensure justice is done to each individuals’ story at later stages of analysis [51]. The QUAGOL method preserves individual narratives by first constructing narrative interview reports and conceptual interview schemes that stay close to participants’ own words. It then combines detailed within-case analysis with across-case analysis, ensuring that findings are both grounded in individual experiences and informed by patterns across participants. Conceptual interview schemes were compared across interviews to identify emergent themes. A preliminary coding scheme was developed and applied by one researcher (WA), with subsequent verification by a second researcher (DR). As new codes emerged from subsequent interviews, the coding scheme was refined and applied retrospectively. Concepts were further developed into detailed descriptions encompassing meaning, patterns, dimensions, and characteristics, and integrated into a conceptual framework or storyline. Interpretation and further shaping of the themes was done together with social workers, budget coaches and management from the participating organization. Data analysis was facilitated using Atlas.ti 22 software, and results were reported in accordance with the Standards for Reporting Qualitative Research (SRQR) [52].

## Results

Table 1 summarizes the characteristics of the study participants. The majority of the sample (n = 9) was between 20 and 29 years old. While 22% of the clients resided in supported housing, 78% were in floating outreach, shelter facilities for the homeless, or received budget coaching exclusively from the supported housing organization. This distribution is representative of the target population [53]. Most participants had been receiving financial support from a budget coach for over two years. Only five of the 28 participants were employed, and one client owned a home. The remaining participants rented privately or through the supported accommodation organization. While the majority of clients had previously been in debt, only three had active debts at the time of the interview.

**Table 1:** Characteristics of study sample (*N* = 28).

	Number of clients *n* (%)
Age in years at time of interview *M* (*SD*)	41.15 (14.74)
20-29	9 (33.3%)
30–39	5 (18.5%)
40–49	5 (18.5%)
50–59	5 (18.5%)
60–69	2 (7.4%)
> 70	1 (3.7%)
Gender	
Male	16 (57.1%)
Female	12 (42.9%)
Type of support	
Residential support	6 (22.2%)
Floating outreach	13 (48.1%)
Shelter facilities	2 (7.4%)
Budget coaching^a^	4 (14.8%)
Number of months in care *M* (*SD*)	40.70 (16.66)
0-12	2 (7.4%)
13-24	4 (14.8%)
> 25	21 (77.8%)

Note: from one client no demographic information was available, except the gender

^a^These clients either previously received other types of care and now only have a budget coach, or have only ever received budget coaching from the organization

### The beginning of financial hardship

#### Perceived causes.

Clients reported various, often intertwined causes for their financial problems. Many of these causes are not exclusive to clients but are common challenges, such as experiencing debt after a divorce. While mental health problems are rarely the sole cause of financial hardship, they play a significant role in maintaining or exacerbating these issues. Clients described psychological factors such as emotional instability, depression, borderline personality disorder, autism, loneliness, and manic episodes as contributing to their financial struggles. These psychological issues can lead to impulsive behavior, a lack of financial oversight, and excessive spending. Additionally, the stress of debt can itself contribute to psychological problems, making it even more challenging to solve the issues.

A lack of financial skills was identified by the researchers as the primary and most significant cause of clients’ financial difficulties. A large proportion of clients had never learned how to manage money effectively. They often expressed being ‘bad at organizing things in general’, making it challenging to keep track of income and expenses. This led to impulsive buying behavior and unnecessary expenditures and leads clients into a vicious cycle: they have no overview of their income and expenses, spend too much money, fall into debt, and see no way out.

“I have a compulsion to spend money and that’s a psychological thing. […] For example, with the current corona situation and the lockdown, which has been quite stressful for me, it’s made the urge to spend money even stronger. And then I’m glad there’s someone else managing my finances so I can’t access it.” – P14

“Yes, well, I’m a single person. And when I first started living on my own, I really began accumulating debt because I couldn’t manage money. If I was €1,000 in the red on one account, I would just use another account. I would simply go to another bank and open another account. And it eventually got so out of hand that I had around €2,000 or €3,000 in debt.” – P10

Relationship breakdowns, including divorce and abuse, were narrated as a significant impact on the financial situation of clients, and were often an important cause for a debt cycle, i.e., a recurring pattern where individuals accumulate debt, struggle to repay it, and find themselves further in debt due to interest payments or fees. Several clients indicated that financial problems started with the relationship, and ending the relationship made it even worse.

“I had a relationship and got into financial trouble because of it. When we broke up, I got even deeper into financial trouble. Eventually, I got debt assistance. It was resolved with a lot of difficulty, but the debt assistance in [place] was very poorly organized, so it took about eight years before I was somewhat out of it. And I came out of debt assistance with new debts. There were some mistakes … In short, I came out of debt assistance with debts and actually also severely depressed and in bad shape.” – P4

Support from friends and family can serve as both a protective factor and a potential contributor to financial hardship. While most clients indicated having some level of social support, it was often limited. Those who identified social support as a contributing factor to their financial problems generally described receiving financial assistance from parents or family for large expenses or specific incidents. In some cases, this reliance on others may have hindered the development of independent financial management skills. Additionally, two clients reported debts directly resulting from abuse or exploitation by friends or partners.

“But yeah, I was always deceived by a lot of people, including women, they would say, ‘We’ll take care of your worries’ and ‘We’ll help you.’ Yeah, so I thought to myself, ‘Okay, those people will help you.’ But it only got worse and worse of course. And then you start drinking a lot more naturally.” – P15

### Experiences of financial hardship

#### Stress.

For many clients, financial hardship was a constant preoccupation, consuming their thoughts and affecting their daily lives, as ‘everything costs money’. They were frequently concerned with making ends meet and paying off debts, leading to significant stress and tension.

#### Short-term orientation.

Many clients mentioned resorting to impulsive decision-making, such as excessive spending or avoidance behavior. This behavior often led to a vicious cycle of debt, often fueled by anxiety, stress, and feelings of shame.

#### Shame.

A sense of failure towards oneself and one’s family is also accompanied by feelings of guilt, leading to social withdrawal. Sharing their financial struggles after years of keeping them secret was often difficult and vulnerable due to the associated guilt and shame.

“But look, at a certain point you also have to swallow your pride. Look, I come from a family, I don’t want to say they’re swimming in money, but they could at least dip their toes in. So it was also a kind of defeat for me that I let things get this far, you know? In our family, it’s not normal to have debts. So I also felt a lot of shame and guilt about letting it get this far.” – P13

#### Impact.

Financial problems had a profound impact on clients’ lives, particularly in their emotional and social well-being, and on their sense of independence. Life events like divorce and job loss led to debt, which in turn exacerbated stress, short-term thinking, and difficulty in resolving issues. This could manifest in experiences of depression, sleep problems, and addiction for some clients. Socially, clients often isolated themselves, fearing judgment and the potential loss of friendships. Conversely, feelings of sadness and loneliness could exacerbate financial problems. For many clients, financial hardship was paralyzing, putting their lives on hold. They were forced to cut back on enjoyable activities, essential expenses, and even basic necessities like maintaining their homes. It undermines their independence and autonomy, leaving them feeling stagnant and merely surviving, unable to pursue personal development, as this requires financial resources. Additionally, clients felt a loss of independence as managing their finances became impossible, necessitating external support.

“Friendships, for example, not daring to meet up with people because you’re afraid they’ll suggest going to McDonald’s while you don’t have money for that. Or having to say no when people go out for a sandwich together, saying, sorry, but I can’t afford that. Someone might understand that once, but when it happens over a long period, it affects your relationships.” – P4

#### Reasons for starting financial support.

Clients expressed several reasons for eventually seeking financial support. Many lacked sufficient funds to cover their living expenses, often resorting to borrowing from one source to repay another. They were also unable to meet their fixed financial obligations. Clients faced unrepayable debts, felt overwhelmed by their financial situation, or recognized the need for change and realized that financial support was the only viable solution. Additionally, many clients lacked the necessary financial skills to manage their finances effectively. Many participants indicated that they only sought assistance when their financial situation had deteriorated to the point where they saw no other options.

“Debts started to accumulate and bailiffs came after me because certain things weren’t being paid due to my ostrich mentality. But at some point, I realized that it couldn’t go on like that. So I talked to a GP, and it wasn’t that I was physically unwell, but mentally it was a mess. Well, I could talk very well with the GP, and they put me in touch with a social nurse, who then connected me with [organization 2].” – P13 talking about how the start of financial support went

### The role and impact of financial support on financial stability and self-management

#### Tailored financial support.

Budget coaching was typically initiated by social workers, reflecting a proactive and integrated approach to financial support. This was a voluntary process for most clients, with the exception of those entering sheltered facilities, where it was often a standard component of the support offered as clients entering these services presented with proportionally more financial problems. In some cases, family members facilitated the initial contact with budget coaches. Clients frequently reported that the initial phase of budget management was challenging. Sharing their financial situation and relinquishing control of their finances could be confronting, especially due to feelings of shame. However, most clients ultimately expressed satisfaction with seeking help. The majority of clients were referred to budget coaches by other professionals, primarily within the organization, highlighting the collaborative nature of support within this setting. It is important to note that budget coaches operate independently from regular support staff, such as personal and residential support workers, offering specialized financial expertise.

Clients valued the personalized guidance provided by budget coaches. The coaches tailored the frequency of contact, the level of financial management support, and savings goals to meet each client’s individual needs. Initially, budget coaches typically managed all fixed expenses, beginning with a comprehensive overview of income and expenditures. Subsequently, coaches assisted clients in building savings funds, applying for allowances, and contacting relevant agencies. Over time, clients learned to take increasing responsibility for their finances as much as possible, with ongoing support from the budget coach.

“In the beginning [of the budget coaching trajectory] they took over everything and she got everything in order so that money came in and went out. […] So now I can see in an online program what comes in, what goes out, and what is in the account and in which pot there is what. That was added later. We have now started with me having more money in my own account again and seeing if it stays there or if I then feel again that I have to spend it. I’m now at a point where I’m trying to see if I can make sure I don’t have that anymore. So we are making progress.” – P4

Budget coaches were accessible through various channels, including face-to-face meetings, apps, email, and phone. Regular appointments were often supplemented by ad-hoc contact for urgent matters. Clients generally reported positive experiences with communication with their budget coaches. While some clients found it difficult to transition to a new budget coach if their established budget coach left or their case was transferred, due to established relationships and feelings of shame, they generally noted little difference in working methods and a smooth transition process.

#### Empowering financial stability and self-management.

Budget coaching contributed to clients’ financial stability in several ways. Clients appreciated the management of fixed expenses, the organization of their finances, and the increased oversight of income and expenses. Many clients also benefited from assistance with paying off debts, which they would have struggled to achieve on their own. Clients frequently expressed that budget coaches accomplished more than they could have done independently, noting that agencies (e.g., health insurance or debt collection agencies) were more responsive and cooperative when dealing with their budget coach.

The extent to which clients acquire financial skills varied. Some clients were already relatively independent but lacked the ability to manage their finances due life events or mental health issues. Others reported significant improvements in their financial skills, including budgeting, reducing impulsive spending, enhancing financial oversight, and improving their ability to plan and save.

“I have become much more aware of what things cost and when you spend money. That too, definitely. So I started doing very simple, logical things. I started paying attention to discounts and going to different supermarkets and such. I always just went for the easiest and what I liked best.” - P11

Many clients expressed their intention to manage their finances independently in the future. They were gradually learning to pay more bills and take greater responsibility for their finances. While some clients were actively working towards this goal, others were more hesitant. Whether clients want to phase out budget coaching in the future depends on their individual situation. Some clients desired continued support until their debts were repaid, while others were not yet ready to consider that step. Clients who were already managing their bills independently indicated their confidence in their ability to maintain financial self-management.

Improving financial stability and skills by using specialized professional support had positive effects on clients’ lives, including reduced stress and increased peace of mind. They were often exhausted by their financial struggles, had to ask for help, and it was a great relief that their finances were now managed. This lifted a weight off their shoulders. The sense of stability and continuity provided by financial management allowed clients to focus on other support goals that had previously been challenging. Some clients also reported increased self-confidence and a sense of pride in their accomplishments.

“And then I make sure that my energy bill is deducted earlier because then it stays on my account for a shorter time, which makes me very happy. And that’s also a bit of independence, I think. And then I get feedback that I’m doing very well, and that motivates me enormously.” – P12

Clients emphasized the importance of the relationship with the budget coach in the success of budget coaching. They valued open and clear communication, a sense of connection, and the professional attitude of the budget coach. These factors facilitated discussions about personal financial challenges. Clients appreciated the positive feedback and encouragement provided by their budget coaches, which contributed to feelings of empowerment. The professionalism, punctuality, and client-centered approach of the budget coaches were also highly valued.

“I always had good contact with her and I think that’s important… She was honest with me. Maybe sometimes a bit harsh or a bit painful, but that’s okay. I prefer people who are straightforward with me, who tell it like it is. I can only respect that. I had that with [person] as well and I told her that I felt that way. I appreciated what she did for me and I valued her honesty. I told her that as well.” – P21

## Discussion

This study aimed to investigate the experiences of financial hardship among clients in supported accommodation, focusing on identifying contributing factors, the impact of financial hardship, and support strategies that foster financial stability or self-management. Our findings highlight that financial hardship for these clients is a complex interplay of various factors, including a lack of financial skills, relationship difficulties, stressful life events, and mental health challenges. The resulting stress and anxiety can further exacerbate financial problems and lead to feelings of shame and social isolation. Clients sought financial support primarily due to insufficient funds, an inability to repay debts, and a desire for financial stability. The support provided by budget coaches, involving personalized guidance, assistance with financial management, and the development of financial skills, proved valuable in improving clients’ financial situations, reducing stress, and enhancing their overall well-being.

A significant finding is that clients’ financial difficulties often stem from a complex interplay of various factors, including a lack of financial skills, relationship problems or separations, and mental health issues. Mental health issues are rarely isolated causes but often exacerbate existing problems or are a consequence of financial hardships. Our findings align with those of previous studies. Kealy et al. [54] support the interconnection between mental health issues and financial hardship, while also highlighting the low incomes of clients and the lack of financial skills as primary contributors to financial problems. Forchuk et al. [35] found that participants described how the stress of financial instability worsened their mental health, while their mental illness made it harder to escape poverty. This, in turn, hindered their ability to actively engage in recovery. Additionally, many clients described being stuck in a cycle or trap of poverty that was difficult to escape. Financial hardship is often deeply intertwined with social and economic marginalization, which can further worsen mental health issues [54]. Moreover, the consequences of poverty and mental illness often overlap; symptoms such as complexity, apathy, and depression are frequently observed in both conditions, making it difficult to attribute these symptoms to a single cause [42,55,56]. This complexity underscores the importance for professionals to adopt a contextual approach, recognizing the interconnectedness of individual mental health, societal factors, and financial well-being [55,57].

This interconnectedness and complexity of the issues have several practical implications. Firstly, mental health services should adopt a more integrated approach and improve collaboration and referrals regarding financial support. Secondly, solutions need to address not just individual behaviors (like financial management) but also broader societal factors like education access, employment opportunities (while also acknowledging potential benefit traps), and government policy reform [35].

Our findings highlight the interconnectedness of financial stability and mental health, and underline the normal and general reaction of stress to financial hardship, serving as an important consideration in diagnostics to avoid potential misinterpretation or over-medicalization. The limited effectiveness of traditional mental health interventions in the absence of financial stability underscores this relationship [58,59]. This evidence supports a holistic approach, similar to the ‘Housing First’ model, which prioritizes meeting basic needs before addressing other life domains [60]. Participants in our study reported experiencing increased peace of mind and a greater capacity to work on other support goals once their financial concerns were (temporarily) alleviated. By creating financial stability, individuals felt better equipped to focus on addressing underlying mental health problems and developing coping mechanisms.

The finding that many clients felt ashamed of their financial problems aligns with other studies [10,35,43,61]. The stigma associated with financial difficulties is often rooted in the belief that such problems are self-inflicted, easily resolved, or stem from their mental illness. This perception is reinforced by studies indicating that individuals experiencing financial hardship may face stigma from family and friends, leading them to conceal their problems [35]. Contrary to the belief that debt and mental illness go hand in hand, our findings suggest that most clients’ financial difficulties were attributable to factors such as adverse life events, relationship issues or lack of financial skills, rather than their mental health conditions. The stigma surrounding financial problems has far-reaching consequences, besides delayed help seeking. Financial struggles contribute to social isolation and feelings of stigma, limiting individuals’ ability to engage in social activities or maintain relationships, thus reducing both their economic and emotional well-being [43]. Financial resources provide access to social networks that can facilitate employment and social mobility [19]. This underscores the need for earlier identification of financial difficulties and easy access to solutions. Current practices often involve addressing financial issues only when they have reached a crisis point. To address this gap, regular financial assessments could be incorporated into treatment plans, using brief screening tools to identify potential financial concerns, i.e., be considered an integral part of comprehensive care. In addition, our findings suggest that coping mechanisms deserve further investigation, particularly how maladaptive strategies such as avoidance may hinder financial self-management and how professionals can help clients develop more adaptive responses to financial stress.

Our findings indicate that clients expressed a high level of satisfaction with the support provided, especially by budget coaching. This suggests that a combination of target group knowledge and specialized financial expertise is effective for individuals in supported accommodation. Given the significant overlap in client populations, the positive outcomes of this intervention could be extended to outpatient mental health care settings. Future research should investigate the efficacy of similar financial guidance programs for individuals receiving treatment. In addition, future studies could explore these themes using quantitative methods, for example by assessing perceived support from budget and debt counselling services in a larger, representative sample, or by examining associations between financial literacy and mental health outcomes. Such research would help validate and expand upon the insights from this qualitative study and inform the development of scalable interventions.

The literature offers various approaches to assist individuals experiencing poverty, debt, or financial hardship. Given the lower savings rates among individuals with mental health conditions, asset-building programs have been implemented in countries such as the United States [62,63]. Other interventions focus on developing money management skills. While limited research exists, studies on veterans have shown that longer-term training programs combining financial management and education are more effective than single-session psychoeducation [39,64]. Our finding that building a strong working relationship with the budget coach, with room for shared decision-making, was beneficial aligns with rehabilitation and citizenship-oriented systems of care [65,66]. Clients should be actively involved in decision-making regarding their financial management and supported in developing their own skills, fostering a sense of empowerment and promoting recovery [67,68].

### Strengths and limitations

Our study has provided deeper insights into the factors contributing to financial difficulties among clients, how these are experienced, and what supports clients in achieving greater financial stability. Another strength is the inclusion of a large and diverse group of clients within a Dutch supported accommodation organization, providing a comprehensive picture of the financial struggles faced by this specific population and the factors that assist them. The use of the Qualitative Analysis Guide of Leuven (QUAGOL) is an additional strength. Its combination of within-case and across-case analyses helped ensure that findings were both grounded in individual experiences and informed by patterns across participants, while preserving the integrity of participants’ narratives. A limitation of the method is that translating rich narrative data into conceptual schemes requires careful analytical work; if done too rigidly or selectively, there is a risk of focusing too much on details and losing sight of the essence, or being biased by other cases [51].

The study also has broader limitations. Firstly, the recruitment process focused on clients already receiving financial support, meaning we primarily captured the experiences of those actively engaged in addressing existing financial challenges. This approach may have excluded individuals who, while not currently in debt or receiving formal financial assistance, still experience financial vulnerability or struggle with financial management. To gain a more comprehensive understanding of financial difficulties and their broader impact across the entire client population in supported accommodation settings in the Netherlands, future studies should consider including all clients, regardless of their current financial support status. Secondly, participant selection was facilitated through professionals and was voluntary, potentially introducing (self-)selection bias. Finally, the study’s focus on a specific supported accommodation organization in the Netherlands may limit the generalizability of findings to other settings and countries.

## Conclusions

The study has highlighted that financial difficulties among clients in supported accommodation are often complex and multifaceted. The onset of financial hardship can be traced to various factors, including limited financial skills, relationship problems, and mental health issues. These factors can create a vicious cycle of debt and stress. The study highlights the importance of tailored financial support that not only addresses immediate financial needs but also focuses on the underlying causes and empowering clients to become more financially independent. It underscores the importance of addressing financial concerns within mental health support and treatment and considering broader social inequities as contributing factors to both financial and mental health challenges within the Dutch context. By gaining a deeper understanding of the complex dynamics of financial difficulties in this population, effective interventions can be developed and enhance the financial resilience of vulnerable individuals in supported accommodation.

## Supporting information

S1 Appendix Interview guide. (PDF)

## References

[1] Ahnquist J, Wamala SP. Economic hardships in adulthood and mental health in Sweden. The Swedish National Public Health Survey 2009. BMC Public Health. 2011;11:788. doi: 10.1186/1471-2458-11-788 21989478 PMC3206480

[2] Borras L, Mohr S, Boucherie M, Dupont-Willemin S, Ferrero F, Huguelet P. Patients with schizophrenia and their finances: how they spend their money. Soc Psychiatry Psychiatr Epidemiol. 2007;42(12):977–83. doi: 10.1007/s00127-007-0257-1 17901909

[3] Eklund M. Work status, daily activities and quality of life among people with severe mental illness. Qual Life Res. 2009;18(2):163–70. doi: 10.1007/s11136-008-9431-5 19125354

[4] Evans JD, Wright DE, Svanum S, Bond GR. Psychiatric disorder and unmet service needs among welfare clients in a representative payee program. Community Ment Health J. 2004;40(6):539–48. doi: 10.1007/s10597-004-6127-3 15672692

[5] Jenkins R, Bebbington P, Brugha T, Bhugra D, Farrell M, Coid J. Mental disorder in people with debt in the general population. Public Health Med. 2009;6(3):88–92.

[6] Jenkins R, Bhugra D, Bebbington P, Brugha T, Farrell M, Coid J, et al. Debt, income and mental disorder in the general population. Psychol Med. 2008;38(10):1485–93. doi: 10.1017/S0033291707002516 18184442

[7] Marum G, Clench-Aas J, Nes RB, Raanaas RK. The relationship between negative life events, psychological distress and life satisfaction: a population-based study. Qual Life Res. 2014;23(2):601–11. doi: 10.1007/s11136-013-0512-8 24026629

[8] Perese EF. Stigma, Poverty, and Victimization: Roadblocks to Recovery for Individuals With Severe Mental Illness. J Am Psychiatr Nurses Assoc. 2007;13(5):285–95. doi: 10.1177/1078390307307830

[9] Walker R, Kyomuhendo GB, Chase E, Choudhry S, Gubrium EK, Nicola JY, et al. Poverty in Global Perspective: Is Shame a Common Denominator? J Soc Pol. 2013;42(2):215–33. doi: 10.1017/s0047279412000979

[10] Spivak S, Cullen B, Eaton WW, Rodriguez K, Mojtabai R. Financial hardship among individuals with serious mental illness. Psychiatry Res. 2019;282:112632. doi: 10.1016/j.psychres.2019.112632 31690462

[11] Lorant V, Croux C, Weich S, Deliège D, Mackenbach J, Ansseau M. Depression and socio-economic risk factors: 7-year longitudinal population study. Br J Psychiatry. 2007;190:293–8. doi: 10.1192/bjp.bp.105.020040 17401034

[12] Evans K. Treating financial difficulty - the missing link in mental health care? J Ment Health. 2018;27(6):487–9. doi: 10.1080/09638237.2018.1520972 30417733

[13] Sapolsky R. Sick of poverty. Sci Am. 2005;293(6):92–9. doi: 10.1038/scientificamerican1205-92 16323696

[14] Davis LV, Hagen JL. Stereotypes and Stigma: What’s Changed for Welfare Mothers. Affilia. 1996;11(3):319–37. doi: 10.1177/088610999601100304

[15] Jung J, Mukherjee K, Brown M, Sadigh G. Association between financial hardship and psychological burden and the role of social and mental health support: An observational study. Medicine (Baltimore). 2024;103(28):e38871. doi: 10.1097/MD.0000000000038871 38996144 PMC11245238

[16] Fitch C, Hamilton S, Bassett P, Davey R. The relationship between personal debt and mental health: a systematic review. Mental Health Review Journal. 2011;16(4):153–66. doi: 10.1108/13619321111202313

[17] Jungmann N, Wesdorp P. Mobility Mentoring: Hoe inzichten uit de hersenwetenschap leiden tot een betere aanpak van armoede en schulden. Den Haag. 2017.

[18] Topor A, Andersson G, Denhov A, Holmqvist S, Mattsson M, Stefansson C-G, et al. Psychosis and poverty: Coping with poverty and severe mental illness in everyday life. Psychosis. 2013;6(2):117–27. doi: 10.1080/17522439.2013.790070

[19] Harper A, Clayton A, Bailey M, Foss-Kelly L, Sernyak MJ, Rowe M. Financial Health and Mental Health Among Clients of a Community Mental Health Center: Making the Connections. Psychiatr Serv. 2015;66(12):1271–6. doi: 10.1176/appi.ps.201400438 26234330

[20] Bond GR. Supported employment: evidence for an evidence-based practice. Psychiatr Rehabil J. 2004;27(4):345–59. doi: 10.2975/27.2004.345.359 15222147

[21] de Lange A, Michon HWC, Knispel A, Hulsbosch L, Twisk JWR, Kroon H. It is not only competitive employment that counts: Findings from a longitudinal panel of people with severe mental illness. Psychiatr Rehabil J. 2022;45(3):266–72. doi: 10.1037/prj0000524 35771516

[22] Marwaha S, Johnson S. Schizophrenia and employment - a review. Soc Psychiatry Psychiatr Epidemiol. 2004;39(5):337–49. doi: 10.1007/s00127-004-0762-4 15133589

[23] Wahlbeck K, Cresswell-Smith J, Haaramo P, Parkkonen J. Interventions to mitigate the effects of poverty and inequality on mental health. Soc Psychiatry Psychiatr Epidemiol. 2017;52(5):505–14. doi: 10.1007/s00127-017-1370-4 28280872

[24] Central Bureau for Statistics. Debt problems in focus: Households with registered problematic debts 2015-2021 [Schuldenproblematiek in beeld: Huishoudens met geregistreerde problematische schulden 2015-2021]. Den Haag: 2022.

[25] Holkar M. Seeing through the fog. Money and Mental Health Policy Institute. 2017.

[26] Skapinakis P, Weich S, Lewis G, Singleton N, Araya R. Socio-economic position and common mental disorders. Longitudinal study in the general population in the UK. Br J Psychiatry. 2006;189:109–17. doi: 10.1192/bjp.bp.105.014449 16880479

[27] Killaspy H. Supported accommodation for people with mental health problems. World Psychiatry. 2016;15(1):74–5. doi: 10.1002/wps.20278 26833612 PMC4780284

[28] McPherson P, Krotofil J, Killaspy H. Mental health supported accommodation services: a systematic review of mental health and psychosocial outcomes. BMC Psychiatry. 2018;18(1):128. doi: 10.1186/s12888-018-1725-8 29764420 PMC5952646

[29] Chilvers R, Macdonald GM, Hayes AA. Supported housing for people with severe mental disorders. Cochrane Database Syst Rev. 2006;2006(4):CD000453. doi: 10.1002/14651858.CD000453.pub2 17054130 PMC7032537

[30] Bitter NA, Roeg DPK, van Nieuwenhuizen C, van Weeghel J. Identifying profiles of service users in housing services and exploring their quality of life and care needs. BMC Psychiatry. 2016;16(1):419. doi: 10.1186/s12888-016-1122-0 27881159 PMC5120432

[31] de Heer-Wunderink C, Visser E, Caro-Nienhuis A, Sytema S, Wiersma D. Supported housing and supported independent living in the Netherlands, with a comparison with England. Community Ment Health J. 2012;48(3):321–7. doi: 10.1007/s10597-011-9381-1 21246274 PMC3371186

[32] Bengtsson-Tops A, Hansson L. Clinical and social needs of schizophrenic outpatients living in the community: the relationship between needs and subjective quality of life. Soc Psychiatry Psychiatr Epidemiol. 1999;34(10):513–8. doi: 10.1007/s001270050169 10591810

[33] Jansen JL, Bruggeman R, Kiers HAL, PHAMOUS-investigators, Pijnenborg GHM, Castelein S, et al. Financial dissatisfaction in people with psychotic disorders - A short report on its prevalence and correlates in a large naturalistic psychosis cohort. J Psychiatr Res. 2024;170:302–6. doi: 10.1016/j.jpsychires.2023.12.039 38185076

[34] Priebe S, Saidi M, Want A, Mangalore R, Knapp M. Housing services for people with mental disorders in England: patient characteristics, care provision and costs. Soc Psychiatry Psychiatr Epidemiol. 2009;44(10):805–14. doi: 10.1007/s00127-009-0001-0 19277440

[35] Forchuk C, Montgomery P, Rudnick A, Lahey P, Cohen B, Schofield R, et al. Poverty Trajectories Experienced by Persons with Mental Illness. Journal of Poverty. 2016;21(3):247–64. doi: 10.1080/10875549.2016.1186772

[36] Conrad KJ, Lutz G, Matters MD, Donner L, Clark E, Lynch P. Randomized trial of psychiatric care with representative payeeship for persons with serious mental illness. Psychiatr Serv. 2006;57(2):197–204. doi: 10.1176/appi.ps.57.2.197 16452696

[37] Rosen MI, Ablondi K, Black AC, Serowik KL, Rowe M. Pathways to assignment of payees. Community Ment Health J. 2014;50(3):270–4. doi: 10.1007/s10597-013-9629-z 23765182 PMC3858572

[38] Tsai J, Ablondi K, Payne K, Rosen MI, Rosenheck RA. Recovery-oriented money management for homeless veterans: a feasibility study. Am J Psychiatr Rehabil. 2019;22(3):147–67.

[39] Elbogen EB, Hamer RM, Swanson JW, Swartz MS. A Randomized Clinical Trial of a Money Management Intervention for Veterans With Psychiatric Disabilities. Psychiatr Serv. 2016;67(10):1142–5. doi: 10.1176/appi.ps.201500203 27181733

[40] Lusardi A, Mitchell OS. FINANCIAL LITERACY AROUND THE WORLD: AN OVERVIEW. J Pension Econ Financ. 2011;10(4):497–508. doi: 10.1017/S1474747211000448 28553190 PMC5445931

[41] OECD. PISA 2018 assessment and analytical framework. Paris: OECD. 2019.

[42] Elbogen EB, Tiegreen J, Vaughan C, Bradford DW. Money management, mental health, and psychiatric disability: a recovery-oriented model for improving financial skills. Psychiatr Rehabil J. 2011;34(3):223–31. doi: 10.2975/34.3.2011.223.231 21208861

[43] Caplan MA. Financial coping strategies of mental health consumers: managing social benefits. Community Ment Health J. 2014;50(4):409–14. doi: 10.1007/s10597-013-9674-7 24346222

[44] McPherson P, Krotofil J, Killaspy H. What Works? Toward a New Classification System for Mental Health Supported Accommodation Services: The Simple Taxonomy for Supported Accommodation (STAX-SA). Int J Environ Res Public Health. 2018;15(2):190. doi: 10.3390/ijerph15020190 29364171 PMC5858263

[45] de Heer-Wunderink C. Successful community living: a ‘utopia’? A survey of people with severe mental illness in Dutch regional institutes for residential care. Groningen: Groningen. 2012.

[46] Farkas M, Coe S. From Residential Care to Supportive Housing for People With Psychiatric Disabilities: Past, Present, and Future. Front Psychiatry. 2019;10:862. doi: 10.3389/fpsyt.2019.00862 31849724 PMC6893903

[47] Friesinger JG, Topor A, Bøe TD, Larsen IB. Studies regarding supported housing and the built environment for people with mental health problems: A mixed-methods literature review. Health Place. 2019;57:44–53. doi: 10.1016/j.healthplace.2019.03.006 30959400

[48] Albers W, Roeg D. Financial well-being in supported accommodation: an analysis of the nature and extent of clients’ financial problems and support strategies. Mental Health and Social Inclusion. 2024;29(7):24–34. doi: 10.1108/mhsi-04-2024-0053

[49] Ritchie J, Lewis J, Nicholls CM, Ormston R. Qualitative Research Practice: A Guide for Social Science Students and Researchers. Thousand Oaks, CA: Sage. 2013.

[50] Keen S, Lomeli-Rodriguez M, Joffe H. From Challenge to Opportunity: Virtual Qualitative Research During COVID-19 and Beyond. Int J Qual Methods. 2022;21:16094069221105075. doi: 10.1177/16094069221105075 35692956 PMC9167989

[51] Dierckx de Casterlé B, Gastmans C, Bryon E, Denier Y. QUAGOL: a guide for qualitative data analysis. Int J Nurs Stud. 2012;49(3):360–71. doi: 10.1016/j.ijnurstu.2011.09.012 21996649

[52] O’Brien BC, Harris IB, Beckman TJ, Reed DA, Cook DA. Standards for reporting qualitative research: a synthesis of recommendations. Acad Med. 2014;89(9):1245–51. doi: 10.1097/ACM.0000000000000388 24979285

[53] Roeg D, Bredenoort M. Gaten in de landelijke registraties voor het beschermd en begeleid wonen: een vergelijking van de bestaande monitors. Amersfoort: Valente. 2024.

[54] Kealy D, Spidel A, Sandhu S, Kim D, Izbicki A. Financial concerns and symptom distress among psychiatric outpatients. JPMH. 2018;17(3):105–13. doi: 10.1108/jpmh-12-2017-0041

[55] Topor A, Ljungqvist I, Strandberg EL. Living in poverty with severe mental illness coping with double trouble. Nordic Social Work Research. 2016;6(3):201–10. doi: 10.1080/2156857x.2015.1134629

[56] Jiménez-Solomon O, Irwin G, Melanie W, Christopher W. When money and mental health problems pile up: The reciprocal relationship between income and psychological distress. SSM Popul Health. 2024;25:101624. doi: 10.1016/j.ssmph.2024.101624 38380052 PMC10876910

[57] Priebe S, Burns T, Craig TKJ. The future of academic psychiatry may be social. Br J Psychiatry. 2013;202(5):319–20. doi: 10.1192/bjp.bp.112.116905 23637104

[58] Cohen A, Houck PR, Szanto K, Dew MA, Gilman SE, Reynolds CF 3rd. Social inequalities in response to antidepressant treatment in older adults. Arch Gen Psychiatry. 2006;63(1):50–6. doi: 10.1001/archpsyc.63.1.50 16389196

[59] Areán PA, Gum A, McCulloch CE, Bostrom A, Gallagher-Thompson D, Thompson L. Treatment of depression in low-income older adults. Psychol Aging. 2005;20(4):601–9. doi: 10.1037/0882-7974.20.4.601 16420135

[60] Tsemberis S, Gulcur L, Nakae M. Housing First, consumer choice, and harm reduction for homeless individuals with a dual diagnosis. Am J Public Health. 2004;94(4):651–6. doi: 10.2105/ajph.94.4.651 15054020 PMC1448313

[61] Barnes MC, Donovan JL, Wilson C, Chatwin J, Davies R, Potokar J, et al. Seeking help in times of economic hardship: access, experiences of services and unmet need. BMC Psychiatry. 2017;17(1):84. doi: 10.1186/s12888-017-1235-0 28253879 PMC5335839

[62] Cook JA, Mueser KT. Economic security: an essential component of recovery. Psychiatr Rehabil J. 2013;36(1):1–3. doi: 10.1037/h0094739 23477642

[63] Burke-Miller JK, Swarbrick MA, Carter TM, Jonikas JA, Zipple AM, Fraser VV, et al. Promoting self-determination and financial security through innovative asset building approaches. Psychiatr Rehabil J. 2010;34(2):104–12. doi: 10.2975/34.2.2010.104.112 20952363

[64] Rosen MI, Carroll KM, Stefanovics E, Rosenheck RA. A randomized controlled trial of a money management-based substance use intervention. Psychiatr Serv. 2009;60(4):498–504. doi: 10.1176/ps.2009.60.4.498 19339325 PMC2831204

[65] Rowe M, Serowik KL, Ablondi K, Wilber C, Rosen MI. Recovery and money management. Psychiatr Rehabil J. 2013;36(2):116–8. doi: 10.1037/h0094982 23750764 PMC3992283

[66] Jordan G, Mutschler C, Kidd SA, Rowe M, Iyer SN. Making the case for citizenship-oriented mental healthcare for youth in Canada. JPMH. 2023;22(1):3–11. doi: 10.1108/jpmh-06-2022-0055

[67] Bitter N, Roeg D, van Assen M, van Nieuwenhuizen C, van Weeghel J. How effective is the comprehensive approach to rehabilitation (CARe) methodology? A cluster randomized controlled trial. BMC Psychiatry. 2017;17(1):396. doi: 10.1186/s12888-017-1565-y 29228919 PMC5725818

[68] Wilken JP, Hollander Dd. Handboek steunend relationeel handelen. Werken aan herstel en kwaliteit van leven. 2022.

