# Supplementary material for: Understanding financial hardship and financial recovery among clients in supported accommodation services
Source: PLoS One. 2025 Oct 9;20(10):e0334211. doi: 10.1371/journal.pone.0334211 (PMC12510587; doi:10.1371/journal.pone.0334211)
Supplement: S1 Appendix — (PDF) [file pone.0334211.s001.pdf]

S1 Appendix. Interview guide

Financial problems and solutions among supported accommodation clients

PART 1: Introduction and background

Goal: Getting conversation going and gaining insight into the client's (financial) situation

1. What does the support you receive at Kwintes look like?
2. Can you tell me something about your financial situation?
3. How do you find managing your finances?
4. Can you tell me what the reason is you receive financial support?
5. How did you experience that, that period, asking for help?

PART 2: Impact financial problems

Goal: to understand whether financial problems have an impact on the client’s life and how.

6. How does having financial problems affect you?

PART 3: Financial support

Goal: Getting a description of the financial support a client receives and the client’s experiences with this support

7. Can you tell me something about the help you get with your finances?
8. What does the support you receive from Kwintes look like?
9. How do you feel about the support you receive on your finances?
10. How do you experience it, how do you feel about it?
11. Do you benefit from the financial support?
12. What do you think is important in the financial support?
13. Can you talk a little about your relationship with your personal support worker or budget coach (who helps with your finances)?
14. How do you feel about the contact?

PART 4: (Financial) recovery and future

Goal: To learn how clients look to the future and how financial support affects it. Also: Winding down, back to everyday life, last topic.

15. What are your expectations for the future when it comes to your finances?
16. What are your wishes for the future when it comes to your finances?

PART 5: Closure
